# Predictors of mothers’ home cord care, breastfeeding, and thermoregulation practices for newborns in a South-Eastern State, Nigeria

**DOI:** 10.1186/s12884-025-07705-x

**Published:** 2025-05-22

**Authors:** Chika Chioma Harriet Odira, Blessing Tochukwu Onyeje, Edith Anulika Udeogalanya, Oluwaseyi Isaiah Olabisi, Deborah Tolulope Esan

**Affiliations:** 1 Department of Nursing Science, Nnamdi Azikiwe University, Awka, Anambra State Nigeria; 2 Department of Nursing Science, Chukwuemeka Odumegwu Ojukwu University, Igbariam, Anambra Nigeria; 3 Faculty of Nursing Science, Bowen University, Iwo, Oyo Nigeria

**Keywords:** Newborn care practices, Predictors of mothers’ newborn care, Cord care, Exclusive breastfeeding, Thermoregulation

## Abstract

**Introduction:**

Suboptimal maternal practices in home cord care, breastfeeding, and thermoregulation for newborns pose significant public health concerns, with far-reaching implications for neonatal health. Understanding the determinants influencing these maternal practices is crucial for developing effective interventions.

**Aim:**

This study aimed to identify predictors of mothers' home cord care, breastfeeding, and thermoregulation practices for newborns in a southeastern state in Nigeria.

**Methods:**

A cross-sectional survey of 365 postpartum mothers randomly selected from sampled health facilities in Ihiala Local Government Area was done. Consenting mothers were followed up to their homes. A self-structured, validated, interviewer-administered questionnaire with a reliability score of 0.87 was used for data collection. Appropriate umbilical cord care, breastfeeding, and thermoregulation practices of mothers were evaluated by rating their responses: 1 for YES and 0 for NO. SPSS version 25 was used for data analysis. Data were summarized in tables and charts. Associations between variables were tested using multivariable logistic regression.

**Results:**

The majority (83.3%) of mothers practiced appropriate thermoregulation for their newborns, while only 23.8% and 41.3% practiced exclusive breastfeeding and proper cord care, respectively. The most common reasons given for the poor practice of essential newborn care (ENBC) were ignorance and the influence of family and friends. Predictors of good ENBC practices among the variables tested included place of delivery and educational status of mothers. Place of delivery significantly influenced umbilical cord care, with mothers who delivered in primary health centers showing the lowest odds of practicing proper cord care (AOR = 0.50; 95% CI: 0.28–0.89; *p* = 0.018). Similarly, educational status significantly influenced mothers' thermoregulation practices, with mothers who had only primary education exhibiting the least odd to practicing good thermoregulation (AOR = 0.30; 95% CI: 0.09–0.97; *p* = 0.044).

**Conclusion:**

The study noted sub-optimal ENBC among postnatal mothers specifically: umbilical cord care, practice of exclusive breastfeeding and skin-to-skin contact. The findings underscore the need for targeted, culturally sensitive interventions in Anambra State, Nigeria, particularly focusing on umbilical cord management and breastfeeding.

## Introduction

Proper cord care, breastfeeding, and thermoregulation are fundamental components of essential newborn care, each playing a critical role in safeguarding the health and survival of both mothers and infants [1, 2]. The first 28 days of life are the most vulnerable and critical period for a child’s survival, accounting for approximately 50–70% of infant mortality globally [3]. According to the World Health Organization, 2.3 million children die within the first 28 days of life in 2022, with sub-Saharan Africa experiencing the highest neonatal mortality rate at 27 deaths per 1,000 live births [3]. Nigeria, in particular, faces an even higher rate of 38 per 1,000 live births [4]. Most neonatal deaths linked to inadequate or poorly implemented newborn care practices, especially in the areas of umbilical cord care, thermal regulation, and breastfeeding [5, 6].

Optimal umbilical cord care is essential for preventing infections and promoting proper healing [7]. In Nigeria, umbilical cord infections are responsible for 10–19% of neonatal admissions and contribute to an estimated 30–49% of neonatal deaths [6]. Many studies have demonstrated that application of unclean substances to the cord stump creates a favorable environment for bacterial growth, increasing the risk of sepsis and death [8–11].

The World Health Organization recommends dry cord care and, where infection risk is high, the use of topical antiseptics [11]. Despite these guidelines, the use of unorthodox substances such as hot compresses, cow dung, herbs, ash, and petroleum jelly persists in some communities, elevating the risk of tetanus and sepsis [12–16].

Similarly, breastfeeding provides optimal nutrition and immunological protection for infants, benefiting both children and mothers [17]. However, global exclusive breastfeeding rates remain suboptimal, reported at 44% in 2021, with even lower rates in low- and middle-income countries [18]. Many mothers do not maintain exclusive breastfeeding for the recommended 4–6 months, often due to a lack of support or misinformation [19–21]. Understanding the determinants of breastfeeding practices is crucial for designing effective interventions to improve rates of early initiation and exclusive breastfeeding.

Thermoregulation is another critical aspect of newborn care. Newborns are highly susceptible to hypothermia, which, although rarely a direct cause of death, contributes significantly to neonatal mortality as a comorbidity of severe infections, preterm birth, and asphyxia [22]. Inadequate thermal care practices, such as insufficient skin-to-skin contact or improper swaddling, can lead to hypothermia in newborns [23, 24]. Hypothermia contributes to a substantial proportion of neonatal mortality globally, mostly as a comorbidity of severe neonatal infections, preterm birth, and asphyxia [25].

WHO guidelines emphasize immediate drying and wrapping of the newborn, as well as delayed bathing, to prevent hypothermia. All babies should be exclusively breastfed from birth until 6 months of age. Mothers should be counseled and provided support for EBF at each postnatal contact. Daily chlorhexidine (7.1% chlorhexidine digluconate aqueous solution or gel, delivering 4% chlorhexidine) application to the umbilical cord stump during the first week of life is recommended for newborns who are born at home in settings with high neonatal mortality (30 or more neonatal deaths per 1,000 live births). Clean, dry cord care is recommended for newborns born in health facilities and at home in low neonatal mortality settings. Use of chlorhexidine in these situations may be considered only to replace application of a harmful traditional substance, such as cow dung, to the cord stump. Bathing should be delayed until 24 h after birth. If this is not possible due to cultural reasons, bathing should be delayed for at least 6 h. Appropriate clothing for the baby for the ambient temperature is recommended. This means one to two more layers of than adults and the use of hats/caps. The mother and baby should not be separated and should stay in the same room 24 h a day. Communication and play with the newborn should be encouraged. Immunization should be promoted as per existing WHO guidelines [11].

Certain factors such as maternal age, maternal education, parity, access to healthcare services, family influence, and cultural beliefs have been linked to maternal health behaviors, including newborn care practices [25–27]. Nonetheless, gaps remain in understanding the full range of factors that shape mothers'home care of their newborn in Anambra State.

Given the persistently high rates of neonatal morbidity and mortality in Nigeria, especially in the context of suboptimal home care practices, there is a pressing need for research focused on the determinants of maternal behaviors related to cord care, breastfeeding, and thermal regulation. This study aims to evaluate mothers'home practices related to umbilical cord care, breastfeeding, and thermal regulation for newborns in Anambra State, Nigeria, and to determine the association between selected sociodemographic variables of mothers (age, educational status, occupation, place of delivery, parity) and home care practices for newborns. These variables interact in complex ways to influence maternal health behaviors and care-seeking, underscoring the importance of comprehensive, multifaceted strategies that address these determinants [8, 26, 27]. Gaining insight into the relationship between these factors and maternal adherence to essential newborn care (ENBC) practices is crucial for developing targeted interventions aimed at reducing neonatal mortality in Anambra State.

The Quality Maternal and Newborn Care (QMNC) Framework [28, 29], complemented by a Holistic Framework for Maternal and Newborn Health [30], provides an evidence-based theoretical foundation for evaluating mothers’ home practices related to umbilical cord care, breastfeeding, and thermal regulation, as well as for examining the influence of socio-demographic factors in Anambra State, Nigeria. QMNC and holistic frameworks allow researchers to systematically evaluate mothers’ household practices against established standards, identify gaps between recommended and current practices, analyze how socio-demographic factors influence care behaviors, and make recommendations regarding the development of context-specific, culturally appropriate interventions to improve newborn health outcomes.

## Methods

### Research design

This study employed a cross-sectional survey design for the assessment of mothers’ home care of their neonates.

### Study settings

The study was conducted in Ihiala Local Government Area (LGA), Anambra State, South-East Nigeria. The LGA comprises ten autonomous towns: Ihiala, Okija, Uli, Amorka, Azia, Isseke, Mbosi, Orsumoghu, Lilu, and Ubuluisiuzor [31]. Ihiala Local Government Area hosts numerous healthcare facilities, including Primary Health Centers (PHCs), general hospitals, private hospitals, and maternity homes spread across its ten towns [32]. These facilities provide essential newborn care services such as basic health checks, immunization, and breastfeeding guidance delivered by a collaborative team of health professionals and community health workers to ensure healthy beginnings for infants. However, the availability, timing, and consistency of newborn care vary significantly across different healthcare facilities.

### Target population

This included all women of childbearing age (15–49 years) that delivered their baby in health facilities within the study area. From the 2022 National Population Census projection, the ten towns in Ihiala LGA had a total population of 302,277 [32]. In 2021, women of childbearing age formed 25.6% of the Nigerian population [33]. Therefore, 25.6% of the total population (302,277) is 77,383, forming the population of women of childbearing age in Ihiala LGA.

### Sample size

A sample size of 398 newly delivered mothers was determined using Taro Yamane’s formula, where n = sample size, N = population size, 1 = a constant, and d = level of precision (0.05 at 95% confidence level). *n* = 77383/1 + 77383(0.05)2 = 77383/1 + 193.4575 = 77383/194.4575 = 398.

### Sampling technique

A multistage sampling technique was used. In the first stage, the three senatorial districts comprising Anambra North, Anambra Central, and Anambra South form strata. Anambra South was selected using the simple random sampling method. Secondly, simple random sampling Ihiala L.G.A. from seven Local Government Areas (L.G. As) in Anambra South senatorial district. Thirdly, one village was each randomly selected from the ten autonomous towns in the L.G.A. Fourthly, 10 healthcare facilities, comprising 5 primary health centers, 2 maternity homes, and 3 private hospitals, were sampled from each of the ten villages. Fifthly, newly delivered mothers were consecutively recruited from these facilities and were later followed up to their various homes within the second and third postpartum weeks.

### Inclusion criteria

Nursing mothers (aged 15–49 years) who deliver in health facilities located within the selected villages.

### Exclusion criteria

Mothers who were not resident in Ihiala LGA and mothers who were sick or with sick neonates were excluded from the study.

### Instrument for data collection

Data was collected using self-structured interviewer-administered questionnaire. The questionnaire was designed to collect information regarding the mothers’ socio-demographic characteristics and cord care, breastfeeding, and thermoregulation practices based on the WHO Essential Newborn Care guidelines. Practice of essential newborn care was assessed using “yes/no” questions on various aspects of newborn care (e.g., practice on umbilical cord care, breastfeeding, and thermoregulation). The responses were coded as “1 for correct response (consistent with WHO ENBC guidelines) and 0 for incorrect response (not consistent with WHO ENBC guidelines).” A score was generated for each respondent by dividing the sum of correct responses by the targeted responses and multiplying by 100 [Score = (n/target) × 100 (maximum 100)]. A score of 75% and above was considered good maternal practice, while below 75% was considered poor maternal practice based on similar studies [34–36].

### Validity and reliability of the instrument

To ensure the external and content validity of the instrument, researchers and three experts examined the questionnaire together with the research objectives and hypotheses to ensure that they could accurately measure the intended variables. To ensure data quality, the questionnaire written in English was translated into the local language (Igbo) and back to English and pretested on forty postpartum mothers selected from two healthcare facilities in Ekwusigo L.G.A. of Anambra State, a setting outside the study area. The test–retest method was used to assess the reliability of the instrument. The internal consistency of the items was measured using the Spearman-Brown Coefficient, and an intraclass correlation coefficient of 0.87 was obtained.

Relevant adjustments were made before the questionnaire was finally administered to the research population.

### Data collection methods

Data collection lasted for three months, from June to August 2022. With the approval of the ethics and research committee of the Faculty of Health Sciences and Technology, Nnamdi Azikiwe University Nnewi Campus, and the Anambra State Ministry of Health, the researchers visited the selected health facilities to introduce themselves to the heads of the facilities. The purpose and scope of the study were explained to the healthcare workers and newly delivered mothers in order to gain their consent and cooperation. The healthcare workers were also asked to notify the researchers whenever there is a new delivery so that needed data can be obtained.

Researchers first visited mothers in the postnatal wards within 1 to 3 days after delivery to obtain informed consent and record their contact information. Follow-up visits were then conducted at the mothers’ homes during the second and third weeks postpartum. The initial home visit served primarily for familiarization. During the second visit, researchers evaluated the mothers’ practices regarding umbilical cord care, breastfeeding, and thermal regulation using an interviewer-administered questionnaire designed in accordance with the WHO Essential Newborn Care (ENBC) guidelines [11]. The questionnaire was structured to gather data on the mothers’ socio-demographic profiles as well as their practices related to cord care, breastfeeding, and thermoregulation, in alignment with the WHO Essential Newborn Care (ENC) guidelines. Essential newborn care practices were evaluated through a series of “yes/no” questions addressing key aspects such as umbilical cord care, breastfeeding, and thermoregulation. Each response was coded as “1” if it reflected a correct practice consistent with WHO ENC guidelines, and “0” if it did not align with these recommendations. Each respondent’s score was calculated by dividing the number of correct answers by the total number of targeted questions, then multiplying by 100 to yield a percentage [Score = (number of correct responses/total targeted responses) × 100, with a maximum possible score of 100]. A score of 75% or higher was classified as indicative of good maternal practice, whereas a score below 75% was categorized as poor practice.

Voluntary participation was clearly stated and ensured. Participants’ privacy was protected by implementing strict data security measures to safeguard personal health information. Anonymity and confidentiality were maintained by using identification codes.

## Data analysis

Information obtained was coded and kept secure. Data was entered in a Microsoft Excel sheet, cleaned, and then exported to Statistical Package for Social Sciences (SPSS) version 25 for analysis. Frequency and percentages were used to summarize the results. Multivariable logistic regression analysis was employed to identify key determinants of mothers ‘ENBC practices while controlling for confounding variables. Only variables with *p*-values below 0.25 in the initial analyses were included in the multivariable model. Conclusions were drawn based on the adjusted odds ratios and 95% confidence limits constructed around the estimates. The level of significance was set at *p* < 0.05.

### Ethical considerations

The study was conducted in line with the Declaration of Helsinki. Ethical clearance was obtained from the Research and Ethics Committee, Faculty of Health Sciences and Technology, Nnamdi Azikiwe University, Nnewi Campus, Anambra State. Written administrative permission to proceed with the study was obtained from the Anambra State Ministry of Health, Awka. Approvals were also obtained from the Ihiala Local Government Health Coordinator and the heads of health facilities where mothers were recruited. Verbal consent was obtained from the respondents after explaining to them the purpose of the study. They were assured of the confidentiality of every piece of information collected from them as well as their right and freedom to participate voluntarily or discontinue at any stage of the study without any victimization.

## Results

Out of the 398 mothers sampled, 365 completed the study, and subsequently data collected from them were used in the analysis (representing a 91.7% return rate).

The results in Table 1 indicate that out of 365 respondents, the majority of them, 224(61.4%), were between the ages of 26–34 years; most of the mothers were married 235(64.4%), while the prevailing occupation was trading (42.7%). Also, a higher percentage of the participants had some form of formal education (79.7%) and most delivered their babies in private hospitals.

**Table 1:** Socio-demographic variables of mothers (*n* = 365)

Demographic characteristics	Options	Frequency	Percentage (%)
Age range	18–25 years	37	10.10
	26–34 years	224	61.40
	35 years and above	104	28.50
Marital status	Single	49	13.40
	Married	235	64.40
	Divorced	47	12.90
	Widowed	34	9.30
Occupation	Farming	108	29.60
	Trading	156	42.70
	Civil servant	101	27.70
Highest Educational level	No formal education	74	20.30
	Primary	55	15.10
	Secondary education	175	48.10
	Tertiary education	60	16.50
Place of delivery	Maternity Home	90	24.7
	Primary Health Center	112	30.7
	Private Hospital	162	44.4

**Fig. 1:**
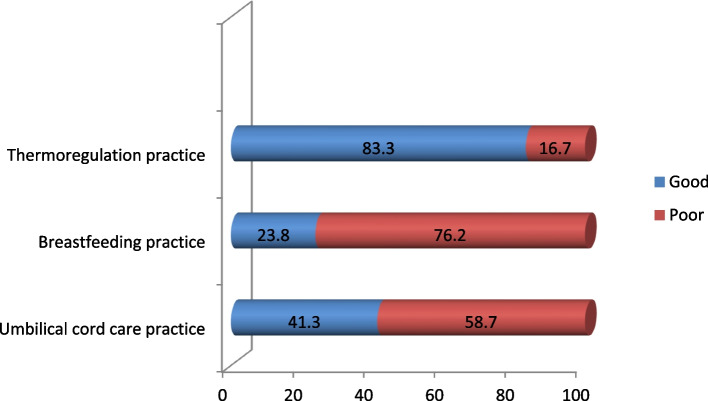
Mean scores of maternal umbilical cord care, breastfeeding, and thermoregulation practices. *Score (%) = *(n/target)* × *100 (maximum 100)*

From Fig. 1 below, it can be deduced that the majority of the mothers observed good thermoregulation practice (83.3%); less than half had good umbilical care practice (41.3%); while 23.8% observed good breastfeeding practice.

Table 2 reveals low rates of exclusive breastfeeding (EBF) in the first week postpartum, with only 27.40% (*n* = 100) of mothers practicing EBF. The 72.60% (*n* = 265) who did not breastfeed exclusively indicated perceived insufficient breast milk (32.45%), husband's opposition (24.53%), occupational constraints (22.65%), influence from in-laws/other women (18.87%), and concerns about breast sagging (2.28%). Only 11.20% (*n* = 41) bathed their babies within 24 h of delivery, citing the need to remove blood (48.78%), prevent body odor (36.58%), and prevent infection (14.64%). Additionally, 6.3% (*n* = 23) used cold water for bathing, believing it strengthens babies (56.52%) and improves sleep (43.48%). Most mothers (75.1%) did not practice skin-to-skin contact, primarily due to lack of awareness (66.79%).

**Table 2:** Probable source of influences on choice of essential newborn care practices among mothers in Anambra State. *n* = 365

Influences on choice of care	Option	Frequency	Percent
Practiced EBF within the first week of delivery	Yes	100	27.40
	No	265	72.60
Reasons for Not practicing EBF	Husband refusal	65	24.53
	Occupation/inadequate time	58	21.87
	Advice of mother in-law/other women	50	18.87
	Avoid sagging of breast	6	2.28
	Breast milk alone insufficient for baby	86	32.45
**Total**		**265**	**100.00**
Delayed bathing baby after 24 h of delivery	Yes	324	88.80
	No	41	11.20
Reasons for bathing baby within 24 h of delivery	To prevent body odour	15	36.58
	To wash out blood from the baby	20	48.78
	To prevent infection	6	14.64
**Total**		**41**	**100.00**
Bathe baby with cold water	Yes	23	6.30
	No	342	93.70
Reasons for bathing baby with cold water	Cold water makes the baby strong	13	56.52
	It improves quality of sleep	10	43.48
**Total**		**23**	**100.00**
Practiced skin-to-skin contact	Yes	91	24.90
	No	274	75.10
Reasons for bathing not practicing skin-to-skin contact with the baby	Never heard about it	183	66.79
	Relatives care for the neonate	63	22.99
	Don’t know how it is done	28	10.22
**Total**		**274**	**100.00**

Although not statistically significant, Table 3 above shows that mothers aged 26–34 years were more than twice as likely to practice good cord care compared to younger mothers aged 18–25 [AOR: 2.16, 95%CI (0.95–4.94)]. Similarly, those with secondary education [AOR: 1.31, 95%CI (0.53–3.27)] and mothers with 3–4 children [AOR: 1.44, 95%CI (0.69–3.01)] showed higher odds of good practices compared to their counterparts with no formal education or fewer children. However, only the delivery location demonstrated a statistically significant association with cord care practices (*p* = 0.018). Mothers who had their delivery in primary healthcare centers were 50% [AOR: 0.50, 95% CI (0.28–0.89)] less likely to practice good umbilical cord care compared to those who delivered in maternity. While mothers who delivered in a private hospital were 37% [AOR: 0.63, 95% CI (0.37–1.06) less likely to practice good umbilical cord care compared to those who delivered in maternity.

**Table 3:** Multivariable logistic regression models of determinants of cord care practices

		**Cord care practice**				
Variable	Category	Poor	Good	COR(95%CI)	AOR(95%CI)	*p*-value
Maternal age (yrs)	18–25 26–34 ≥ 35	16 (7.4%) 129 (60.0%) 70 (32.6%)	21 (14.0%) 95 (63.3%) 34 (22.7%)	1 2.70 (1.25–5.83) 1.52 (0.93–2.47)	1 2.16 (0.95–4.94) 1.46 (0.84–2.54)	0.067 0.179
Educational status	No Formal edu Primary Secondary Tertiary	43 (20.0%) 29 (13.5%) 114 (53.0%) 29 (13.5%)	31 (20.8%) 26 (17.4%) 62 (41.6%) 30 (20.1%)	1 0.70 (0.35–1.39) 0.87 (0.42–1.81) 0.53 (0.29–0.96)	1 1.02 (0.43–2.40) 1.31 (0.53–3.27) 0.61 (0.28–1.31)	0.966 0.560 0.205
Occupation	Farming Trading Civil servant	70 (32.6%) 92 (42.8%) 53 (24.7%)	38 (25.3%) 64 (42.7%) 48 (32.0%)	1 0.60 (0.34–1.05) 0.77 (0.46–1.27)	1 0.62 (0.30–1.28) 0.95 (0.49–1.83)	0.198 0.876
Place of delivery	Maternity Health centre Hospital	62 (28.8%) 69 (32.1%) 84 (39.1%)	28 (18.7%) 43 (28.7%) 79 (52.7%)	1 0.48 (0.28–0.83) 0.66 (0.41–1.08)	1 0.50 (0.28–0.89) 0.63 (0.37–1.06)	0.018* 0.083
Number of children	1–2 3–4 ≥ 5	44 (20.6%) 128 (59.8%) 42 (19.6%)	34 (22.7%) 95 (63.3%) 21 (14.0%)	1 1.55 (0.78–3.08) 1.48 (0.83–2.67)	1 1.44 (0.69–3.01) 1.01 (0.52–1.97)	0.334 0.980

^*^statistically significant, *p* < 0.05

From Table 4, none of the maternal variables appears to be a predictor of good breastfeeding practices, however, mothers aged 35 and older demonstrated higher odds of good breastfeeding practices [AOR: 1.18, 95% CI (0.65–2.14)] compared to younger age groups. Similarly, women with 3–4 children showed an increased likelihood of proper breastfeeding [AOR: 1.44, 95% CI (0.63–3.30)]. Mothers who were traders were 1.49 times [AOR: 2.49, 95% CI (1.02–6.04)] more likely to engage in exclusive breastfeeding than farmers, while those who were civil servants were 1.02 times [AOR: 2.02, 95% CI (0.88–4.65)] more likely breastfeed exclusively than mothers who were farmers.

**Table 4:** Multivariable logistic regression models of determinants of breastfeeding practices in Anambra State

	**Breastfeeding practice**					
Variable	Category	Poor	Good	COR(95%CI)	AOR(95%CI)	*p*-value
Maternal age (yrs)	18–25 26–34 ≥ 35	31 (11.1%) 169 (60.6%) 79 (28.3%)	6 (7.0%) 55 (64.0%) 22 (29.1%)	1 0.61 (0.23–1.64) 1.03 (0.60–1.77)	1 0.61 (0.22–1.70) 1.18 (0.65–2.14)	0.345 0.597
Educational status	No Formal edu Primary Secondary Tertiary	53 (19.1%) 43 (15.5%) 135 (48.6%) 47 (16.9%)	21 (24.4%) 12 (14.0%) 41 (47.7%) 12 (14.0%)	1 1.55 (0.69–3.49) 1.09 (0.44–2.69) 1.19 (0.58–2.45)	1 0.95 (0.34–2.66) 0.58 (0.19–1.77) 0.71 (0.28–1.82)	0.924 0.336 0.473
Occupation	Farming Trading Civil servant	78 (28.0%) 117 (41.9%) 84 (30.1%)	30 (34.9%) 39 (45.3%) 17 (19.8%)	1 1.90 (0.97–3.71) 1.65 (0.87–3.11)	1 2.49 (1.02–6.04) 2.02 (0.88–4.65)	0.044 0.096
Place of delivery	Maternity Health centre Hospital	71 (25.4%) 84 (30.1%) 124 (44.4%)	19 (22.1%) 28 (32.6%) 39 (45.3%)	1 0.85 (0.46–1.58) 1.06 (0.61–1.85)	1 0.74 (0.39–1.42) 0.93 (0.51–1.69)	0.371 0.812
Number of children	1–2 3–4 ≥ 5	56 (20.1%) 172 (61.9%) 50 (18.0%)	22 (25.6%) 51 (59.3%) 13 (15.1%)	1 1.51 (0.69–3.31) 1.14 (0.58–2.26)	1 1.44 (0.63–3.30) 1.26 (0.58–2.74)	0.386 0.556

^*^statistically significant, *p* < 0.05

Table 5 demonstrates that good thermoregulation practices were more likely among civil servants [AOR: 1.92, 95% CI (0.77–4.78)], mothers who delivered at primary health centers [AOR: 2.27, 95% CI (0.99–5.18)], those who received thermoregulation education during antenatal care [AOR: 1.20, 95% CI (0.29–1344.94)], and women with five or more children [AOR: 2.10, 95% CI (0.91–4.84)]. However, only the maternal education level showed a statistically significant association with thermoregulation practices (*p* = 0.044). Lesser odds for good thermoregulation practices were associated with mothers who completed primary education [AOR: 0.30, 95% CI (0.09–0.97)], secondary education [AOR: 0.91, 95% CI (0.23–3.55)] and tertiary education [AOR: 0.81, 95% CI (0.27–2.48)] than with mothers who had no formal education.

**Table 5:** Multivariable logistic regression models of determinants of thermoregulation practices in Anambra State

	**Thermoregulation practice**					
Variable	Category	Poor	Good	COR(95%CI)	AOR(95%CI)	*p*-value
Maternal age (yrs)	18–25 26–34 ≥ 35	7 (11.7%) 40 (66.7%) 13 (21.7%)	30 (9.8%) 184 (60.3%) 91 (29.8%)	1 0.61 (0.22–1.68) 0.66 (0.34–1.29)	1 0.74 (0.24–2.24) 0.58 (0.27–1.26)	0.595 0.171
Educational status	No Formal edu Primary Secondary Tertiary	20 (33.3%) 6 (10.0%) 27 (45.0%) 7 (11.7%)	54 (17.8%) 49 (16.1%) 149 (49.0%) 52 (17.1%)	1 0.36 (0.14–0.93) 1.10 (0.35–3.50) 0.74 (0.31–1.81)	1 0.30 (0.09–0.97) 0.91 (0.23–3.55) 0.81 (0.27–2.48)	0.044* 0.892 0.715
Occupation	Farming Trading Civil servant	27 (45.0%) 16 (26.7%) 17 (28.3%)	81 (26.6%) 140 (45.9%) 84 (27.5%)	1 0.61 (0.31–1.20) 1.77 (0.85–3.69)	1 0.84 (0.35–2.02) 1.92 (0.77–4.78)	0.695 0.161
Place of delivery	Maternity Health centre Hospital	10 (16.7%) 19 (31.7%) 31 (51.7%)	80 (26.2%) 93 (30.5%) 132 (43.3%)	1 1.88 (0.87–4.04) 1.15 (0.61–2.16)	1 2.27 (0.99–5.18) 1.35 (0.67–2.71)	0.052 0.406
Taught thermoregulation care during ANC	No Yes	3 (5.0%) 57 (95.0%)	10 (3.3%) 295 (96.7)	1 1.55 (0.41–5.82)	1 1.20 (0.29–4.94)	0.805
Number of children	1–2 3–4 ≥ 5	19 (31.7%) 26 (43.3%) 15 (25.0%)	59 (19.4%) 197 (64.8%) 48 (15.8%)	1 0.97 (0.45–2.11) 2.37 (1.17–4.81)	1 1.06 (0.45–2.52) 2.10 (0.91–4.84)	0.889 0.082

^*^statistically significant, *p* < 0.05

## Discussion

Fewer than half of the mothers in this study demonstrated proper umbilical cord care practices, significantly increasing the risk of neonatal sepsis and potentially contributing to higher neonatal and infant mortality rates in this population (Fig. 1). Improper cord hygiene increases the odds of neonatal sepsis by 13 times compared to proper care [37]. Furthermore, Obaro et al. noted that poor cord care directly contributes to infections like omphalitis, which can progress to systemic sepsis [38]. A study by Dessalegn et al. reported that neonatal sepsis is a leading cause of mortality in low-resource settings, accounting for 30% of global neonatal deaths [39]. Our findings align with the varying rates of proper umbilical cord care reported across Africa and Nigeria in particular. While Ndikom and colleagues documented 61.4% good practice in Ibadan [13], Abhulimhen-Iyoha et al. found a much lower rate of 20.5% in Edo State [40]. Similarly, Kyololo and Kipkoech reported that only 27% of mothers in one Kenyan study used WHO-recommended chlorhexidine [41]. A systematic review of African studies similarly revealed generally poor cord care practices [2]. The lower rates in Edo State may stem from methodological differences, as that study recruited mothers attending well-baby/immunization clinics with older infants, potentially introducing recall bias. Conversely, the higher rates in Ibadan could be attributed to their larger sample size and focus on mothers with infants specifically between two and eight weeks old.

The first week postpartum is a critical window for establishing breastfeeding. Failure to support and promote exclusive breastfeeding (EBF) during this period undermines efforts to reduce child morbidity and mortality [42]. The finding that the majority of mothers (76.20%) demonstrated poor breastfeeding practices, with 72.60% not practicing exclusive breastfeeding during this critical first week postpartum, carries significant health, social, and economic implications (Fig. 1 and Table 2, respectively). Delayed initiation or absence of breastfeeding in the first 24 h significantly increases infant mortality risk, as this raises the risk of neonatal diseases such as diarrhea, respiratory infections, otitis media, sudden infant death syndrome (SIDS), childhood obesity, type 1 and type 2 diabetes, and leukemia [43]. Suboptimal breastfeeding is associated with lower cognitive outcomes, with any breastfeeding up to six months linked to an approximate 3-point increase in IQ, which has long-term effects on educational attainment and adult earnings [44]. It is also noted that suboptimal breastfeeding is associated with reduced risks of myocardial infarction, hypertension, and stroke in mothers [44]. According to UNICEF, increased rates of these preventable childhood illnesses and maternal health issues place additional burdens on healthcare systems, leading to higher healthcare expenditures and resource use [45].

The most common barriers to exclusive breastfeeding identified by mothers were the belief that breast milk alone was insufficient for their infants, husband’s refusal, inadequate time due to occupation, and advice from mothers-in-law or other women (Table 2). Identifying and addressing these barriers can help improve breastfeeding rates and outcomes [42]. Education, support, and community interventions are essential to promote positive breastfeeding attitudes and practices [42]. Effective interventions should include husbands in prenatal education, as many respondents reported their husbands’ refusal to support exclusive breastfeeding. Mothers should be provided with better support from family members, including husbands, mothers-in-law, and significant others. These results align with a study by Wasti et al., who reported a low prevalence rate of exclusive breastfeeding [21]; however, they differ from the report of Malinga et al., who noted that about 93% of mothers in their study practiced exclusive breastfeeding [1].

Most mothers (83.30%) demonstrated good thermoregulation practices, consistent with previous research findings [1, 5, 46]. However, the low rate of skin-to-skin contact (24.90%), primarily due to lack of awareness, is concerning (Table 2). This aligns with Berhea et al. and Leta’s [5, 46] reports of low thermoregulation knowledge (43.9%) and Kwesiga et al.'s findings of limited Kangaroo Mother Care practice in East-Central Uganda [47]. These results highlight the need for targeted education on the benefits and proper implementation of skin-to-skin contact (SSC). SSC is a proven, evidence-based intervention that supports newborn survival, especially in regions with high neonatal mortality rates. Its absence can contribute to higher rates of preventable newborn deaths [48]. Studies have shown that newborns who do not receive SSC are at higher risk of hospital admission within the first few hours or days of life, primarily due to issues like jaundice and feeding difficulties [49]. Similarly, SSC helps regulate the baby’s heart rate, breathing, and temperature, supporting a smoother transition to life outside the womb. Without SSC, babies are more likely to experience physiological instability [45]. SSC is closely linked to successful breastfeeding initiation, exclusive breastfeeding, and longer breastfeeding duration. Lack of SSC leads to lower breastfeeding rates and increased reliance on formula, which can negatively impact infant nutrition and immunity [50]. Babies who miss out on SSC are more prone to feeding and sucking problems, which can further increase the risk of early hospital readmission and poor growth [45, 49]. The first hour after birth is critical for mother-infant bonding. SSC stimulates the release of oxytocin, fostering emotional connection and maternal behaviors. Lack of SSC can compromise this bonding process, potentially affecting maternal mental health and infant emotional development [45].

UNICEF indicated that SSC enables the transfer of beneficial bacteria from mother to baby, which is important for immune system development. Without it, infants may be more susceptible to infections. SSC has also been shown to lower stress (cortisol) levels in newborns, especially after painful procedures. Babies without SSC may experience higher stress, which can impact development [45]. Since SSC is a low-cost, high-impact intervention, its underutilization represents a missed opportunity for improving newborn outcomes at scale, particularly in resource-limited settings [48]. This study, in line with others, consistently identifies lack of awareness as the leading barrier to SSC implementation [48, 51]. Addressing this gap requires targeted education for both healthcare workers and mothers, as well as clear guidelines and institutional support to make SSC a routine part of newborn care [48, 50, 51].

The study revealed delivery location as a significant predictor of umbilical cord care practices (Table 3), with mothers who delivered in maternity homes associated with better practices than those who delivered in primary health care centers and private hospitals. Possible explanations for this finding include that maternity homes operated by trained midwives might provide more focused and specialized care, leading to better cord care practices [52]. Maternity homes might have more resources, such as guidelines, equipment, and supplies, that support better cord care practices. Maternity homes may prioritize client-centered care, leading to more personalized attention and education on cord care [53]. Primary health care centers and private hospitals may have variable quality of care, resource limitations, or less coordinated care compared to maternity homes designed specifically for maternal waiting and delivery [52, 53].

Although not statistically significant, mothers who were older, had more children, or completed secondary education demonstrated better cord care practices. These trends likely reflect accumulated childcare experience, knowledge from previous births, and greater exposure to evidence-based information in hospital settings. Multiple studies have shown that higher maternal education is associated with better health-seeking behaviors and improved maternal and child health outcomes [2, 54]. In contrast, younger mothers and those with lower education levels are more likely to rely on cultural practices and advice from family and friends. Consistent with this finding, Dessalegn et al., [27] Asiegbu et al. [26] and Abhulimhen-Iyoha et al. [40] found that higher education and greater parity predicted appropriate umbilical cord care. More so, the place of delivery significantly influenced cord care practices, emphasizing the critical role of the immediate postpartum environment and the quality of information provided during this period. Similar findings were reported by Abhulimhen-Iyoha et al. [40] and Malinga et al. [1] However, Abebe et al., [8] observed a different trend. In their study, most women (65.5%) were housewives; a greater number (53%) attended monthly pregnant women’s meetings, and 77.6% had antenatal care follow-ups. Therefore, their study participants had numerous channels through which they accessed beneficial information. Additionally, maternal occupation did not significantly influence cord care practices in this study, similar to what Abebe et al., [8] reported. This observation may be due to the influence of confounders like educational levels, ANC attendance and access to multiple health promotion information. However, Abhulimhen-Iyoha et al. [40] and Malinga et al. [1] found that place of delivery and occupation type were strong determinants of good umbilical cord care.

Furthermore, results show that mothers who are traders and civil servants have higher odds to exclusive breastfeeding practices although not statistically significant (Table 4). Possible explanations for higher odds of exclusive breastfeeding among traders and civil servants compared to farmers could be linked to the fact that traders and civil servants often have more flexible work schedules or structured work environments than farmers, allowing them to take breaks or adjust their routines for breastfeeding or expressing milk [55]. Civil service jobs may provide maternity leave, designated breaks, or even breastfeeding rooms, which facilitate exclusive breastfeeding. In contrast, farming typically involves long hours in the field, far from home and without private or hygienic spaces for breastfeeding or expressing milk [55]. Traders, who may be self-employed or work in markets close to home, can sometimes bring their infants with them, making breastfeeding more feasible compared to farmers working in remote or dispersed locations [57]. They may also have greater access to health information and services, further supporting exclusive breastfeeding [58]. Farming is physically demanding and often requires mothers to be away from their infants for extended periods, making exclusive breastfeeding logistically challenging [56]. In contrast, the less physically demanding and more predictable environments of civil service and trading can make it easier to maintain exclusive breastfeeding routines [57].

It was also observed that higher maternal age and increased parity were associated with improved breastfeeding practices, though not reaching statistical significance. This finding could be attributed to experience garnered with age, having had multiple childbirths, in addition to economic empowerment with an attendant capacity to make better choices. Other studies similarly found that higher parity, [8] better access to essential newborn care information, and consistent antenatal care follow-up [1, 38, 59, 60], all significantly improved the likelihood of proper breastfeeding practices. However, place of delivery and maternal educational status were not significant predictors of good breastfeeding practices in this study; similar to what has been reported by some researchers [1, 46].

Several factors may explain why mothers with primary, secondary, or tertiary education have lower odds of practicing good thermoregulation for their newborns compared to mothers with no formal education (Table 5). More educated mothers may be influenced by modern health beliefs or practices that do not emphasize traditional thermoregulation methods, such as immediate skin-to-skin contact or wrapping the baby warmly. They may perceive these practices as outdated or less necessary, especially if exposed to conflicting information from various sources. A study has found that higher education can sometimes correlate with earlier adoption of formula feeding or early bathing routines, which may disrupt optimal thermoregulation practices. Educated mothers may be more likely to follow hospital routines or advice that do not prioritize traditional thermal care, especially in settings where health worker training is inconsistent [59, 60]. Educated mothers, particularly those who are employed, may return to work sooner or have busier lifestyles, leading to less opportunity for practices like prolonged skin-to-skin contact or exclusive rooming-in, both of which support good thermoregulation.

Mothers with higher education may have greater trust in incubators, heaters, or hospital technology for thermal care, assuming that these replace the need for hands-on thermoregulation practices. Exposure to a wider range of health information, including from the internet and social media, can lead to confusion or selective adoption of practices, sometimes at the expense of basic but effective newborn care routines. In summary, while education is generally associated with better health practices, in some contexts it may lead to lower adherence to traditional or recommended thermoregulation methods due to shifts in beliefs, lifestyle constraints, or trust in alternative approaches. This paradox highlights the importance of context-specific health education and reinforces the value of essential newborn care practices across all education levels [59, 60].

Additionally, occupation type, access to thermoregulation information and delivery in primary health centers all predicted improved practices. These findings align with previous research identifying educational status, maternal occupation and access to essential newborn care information as factors increasing the odds of good thermoregulation practices [1, 46, 59, 60]. However, one contrasting study found no association between maternal education and thermoregulation practices [46], likely due to confounding factors such as high rates of antenatal care attendance (77.6%), institutional deliveries (59.9%) and participation in monthly pregnant mothers'meetings (53%)- all providing alternative channels for disseminating beneficial information.

## Conclusion

This study provides valuable insights into the factors influencing newborn care practices in Anambra State, Nigeria and underscores the need for tailored interventions that promote existing beneficial practices and correct misconceptions surrounding newborn care across diverse populations. The study notes suboptimal ENBC among postnatal mothers, especially umbilical cord care and exclusive breastfeeding practices. The persistence of potentially harmful practices among mothers highlights the need for strategic health education programs that address specific misconceptions and cultural beliefs.

### Recommendations

Public enlightenment on key neonatal care practices-such as exclusive breastfeeding, umbilical cord care, and skin-to-skin contact-should be intensified through mass media, health facility talks, market outreach, and postnatal sessions in religious centers. These efforts should target not only childbearing women but also men, adolescents, and older women to foster community-wide support. Additionally, each state and local government should develop evidence-based neonatal care packages with standardized schedules. Empowering women, families, and communities to adopt and sustain good home-based maternal and newborn care should be a central focus.

### Strengths and limitations of the study

The study ensured representation across Ihiala Local Government Area by sampling one health institution per town, thereby enhancing the accuracy of the findings. Focusing on a single LGA allowed for in-depth engagement, as researchers were able to visit mothers in their homes and interact with them in a familiar environment. However, the results may not be generalizable to other LGAs, as one LGA may not reflect the broader diversity of experiences, contexts and perspectives across the state. Nonetheless, the study can serve as a valuable pilot to refine future research design.

A major limitation to this study was that neonatal care practices were primarily assessed through maternal self-reports, with limited direct observation, which may introduce bias through underreporting or exaggeration. To improve validity and reliability, future studies should triangulate self-reports with observational or objective measures. Despite this limitation, the study offered valuable insights into mothers’ experiences, beliefs, and motivations- providing a foundation for designing targeted interventions and community-based programs.

## Data Availability

The data that support the findings of this study are available from the corresponding author upon request.
